# Supplementary material for: Identification and functional analysis of non-coding regulatory small RNA FenSr3 in Bacillus amyloliquefaciens LPB-18
Source: PeerJ. 2023 May 15;11:e15236. doi: 10.7717/peerj.15236 (PMC10194069; doi:10.7717/peerj.15236)
Supplement: Supplemental Information 4 [file peerj-11-15236-s004.zip › KO/CK-vs-T1_map/map03013.html]

KEGG PATHWAY: RNA transport - Reference pathway


|  |  |
| --- | --- |
| **RNA transport - Reference pathway** |  |

[
Pathway menu
| Organism menu
| Pathway entry
| Show description
| User data mapping
]

RNA transport from the nucleus to the cytoplasm is fundamental for gene expression. The different RNA species that are produced in the nucleus are exported through the nuclear pore complexes (NPCs) via mobile export receptors. The majority of RNAs, such as tRNAs, rRNAs, and U snRNAs, are transported by specific export receptors, which belong to the karyopherin-beta family proteins. A feature of karyopherins is their regulation by the small GTPase Ran. However, general mRNA export is mechanistically different. Nuclear export of mRNAs is functionally coupled to different steps in gene expression processes, such as transcription, splicing, 3'-end formation and even translation.

|  |  |  |
| --- | --- | --- |
| Reference pathway | 184% 150% 122% 100% 82% 67% 55% | 图片下载 |
